# Optimizing Hill Seeding Density for High-Yielding Hybrid Rice in a Single Rice Cropping System in South China

**DOI:** 10.1371/journal.pone.0109417

**Published:** 2014-10-07

**Authors:** Danying Wang, Song Chen, Zaiman Wang, Chenglin Ji, Chunmei Xu, Xiufu Zhang, Bhagirath Singh Chauhan

**Affiliations:** 1 State Key Laboratory of Rice Biology, China National Rice Research Institute, Hangzhou, Zhejiang, China; 2 Key Laboratory of Key Technology on Agricultural Machine and Equipment, South China Agricultural University, Guangzhou, Guangdong, China; 3 Queensland Alliance for Agriculture and Food Innovation, University of Queensland, Queensland, Australia; Instituto de Agricultura Sostenible (CSIC), Spain

## Abstract

Mechanical hill direct seeding of hybrid rice could be the way to solve the problems of high seeding rates and uneven plant establishment now faced in direct seeded rice; however, it is not clear what the optimum hill seeding density should be for high-yielding hybrid rice in the single-season rice production system. Experiments were conducted in 2010 and 2011 to determine the effects of hill seeding density (25 cm×15 cm, 25 cm×17 cm, 25 cm×19 cm, 25 cm×21 cm, and 25 cm×23 cm; three to five seeds per hill) on plant growth and grain yield of a hybrid variety, Nei2you6, in two fields with different fertility (soil fertility 1 and 2). In addition, in 2012 and 2013, comparisons among mechanical hill seeding, broadcasting, and transplanting were conducted with three hybrid varieties to evaluate the optimum seeding density. With increases in seeding spacing from 25 cm×15 cm to 25 cm×23 cm, productive tillers per hill increased by 34.2% and 50.0% in soil fertility 1 and 2. Panicles per m^2^ declined with increases in seeding spacing in soil fertility 1. In soil fertility 2, no difference in panicles per m^2^ was found at spacing ranging from 25 cm×17 cm to 25 cm×23 cm, while decreases in the area of the top three leaves and aboveground dry weight per shoot at flowering were observed. Grain yield was the maximum at 25 cm×17 cm spacing in both soil fertility fields. Our results suggest that a seeding density of 25 cm×17 cm was suitable for high-yielding hybrid rice. These results were verified through on-farm demonstration experiments, in which mechanical hill-seeded rice at this density had equal or higher grain yield than transplanted rice.

## Introduction

Although transplanting has been a major traditional method of rice establishment in most Asian countries, the rising labor cost and developments in rice production technology have improved the desirability of direct-seeded rice [1]. Direct seeding of rice is a low-cost establishment technology. It avoids nursery raising, seedling uprooting, and transplanting, and has the benefits of saving labor, facilitating timely establishment of rice, and earlier crop maturity (by 7–10 days) [2]–[4]. Direct seeding helps in solving the labor scarcity problem, which is now very critical in China’s agricultural development. Simultaneously, the chemical weed control method has made such a switch technically viable. In 2008, for example, 8.3% of the rice-growing area in China was direct-seeded; in some provinces of south China, it was more than 30% [5].

In the south China irrigated rice ecosystem, broadcasting in wet conditions (wet seeding) is the principal method of rice establishment. In this method, pregerminated seeds are sown onto wet (saturated) puddled soils. But, some difficulties are faced in wet seeding. First, no specific varieties have been developed in China for wet seeding. The existing varieties used for transplanting do not appear to be well-adapted for seedling growth in an initially oxygen-depleted micro environment. Second, the extent of laser leveling in China is currently extremely smaller compared with that in other countries (e.g., 50–80% of the rice land in Australia is laser-leveled) [4]. Due to lack of uniform water distribution associated with unevenness of the land, the problem of excess- or no-water-caused yield variability within a field is common, which leads to poor establishment of direct-seeded rice [3], [6]. Third, surface-sown seeds are damaged by birds and rodents. As a result, farmers often resort to the costly practice of increasing the seeding rate for direct-seeded rice, even by two to three times [7]. High seeding rates can result in large yield losses due to excessive number of tillers, increased proportion of ineffective tillers, higher spikelet sterility, and fewer grains per panicle [8]. Moreover, a dense canopy and less ventilation around the plants at high seeding rates can create favorable conditions for diseases (e.g., sheath blight) [9], [10] and insects (e.g., brown planthoppers) and can make plants more prone to lodging [11]. High seeding rates using broadcasting restrict individual plant growth, which is not suitable for the production of high-yielding hybrid rice.

High-yielding hybrid rice, which is crucial to meet expanding food demand, had large panicles and high aboveground biomass [12], [13]. Hybrid rice varieties grow well with proper planting density, but their lodging resistance decreases dramatically under dense seeding [14]. The use of high seeding rate in a broadcast culture may increase the gap between farmers’ potential and actual yield.

Mechanical hill seeding of rice is one of the options used to solve the problem of high seeding rate and uneven plant establishment. Here, rice seeds are sown uniformly in the field at designated spacing and seed number per hill. Combined with a harrow or furrower opener, many hill seeders have land-preparation functions to improve soil evenness. Some hill seeders enable seeding and puddling to be performed simultaneously [15]; others can make furrows and plant the crop at the same time [16]; still others can effectively incorporate seeds into puddled soils at depths of 5–20 mm, which helps decrease losses caused by birds [15]. Even soil conditions and uniform seeding result in an even plant establishment. A well-ventilated micro-environment not only reduces pest and disease incidence but also enhances photosynthesis [17]. In previous studies, lodging resistance of hill-seeded rice was found to be higher than mechanically transplanted rice, row-seeded rice, and broadcast-seeded rice [15], [18].

However, the practical use of mechanical hill seeders had not been seen in China until the 2000s. The adoption of this implement was hampered by the belief that broadcasting is not a suitable method for hybrid rice varieties and both government and farmers still have misgivings about the use of mechanical hill seeding for this purpose. Research is thus imperative to shed light on this technology. There is limited information on hill seeding density for high-yielding hybrid rice varieties, and results for optimum hill seeding density in a single-season rice production system varied from 20 cm×14 cm to 30 cm×20 cm spacing [17], [19]–[21]. It is difficult to draw a concrete conclusion about what hill seeding density is optimum for high-yielding hybrid rice varieties.

The aim of this study was to evaluate the optimum hill seeding density to support the development of mechanical hill seeders for hybrid rice. In this study, 2-year field experiments and 2-year on-farm demonstration experiments were conducted in Zhejiang, China. In the field experiments, a high-yielding hybrid rice variety (Nei2you6) was hill-seeded manually at five seeding densities in two fields with different fertility profiles. The objectives were to evaluate the effects of hill seeding density on plant growth and grain yield, the interrelation between seeding density and soil fertility, and the optimum hill seeding density for high-yielding hybrid rice. In the on-farm demonstrations, three high-yielding hybrid rice varieties were planted using three methods: mechanical hill-seeded with a hill seeder at optimum seeding density, broadcasting, and transplanting following standard farmers’ practice. The objective was to verify the optimum seeding density in the field by comparing the yields of hill-seeded, broadcast, and transplanted rice.

## Materials and Methods

Field experiments and on-farm demonstrations were conducted; field experiments were done in 2010 and 2011 and the on-farm demonstration experiments were carried out in 2012 and 2013. Field experiments were conducted at the experimental farm of the China Rice Research Institute and on-farm demonstration experiments were conducted at the agricultural demonstration base of the China Rice Research Institute in Fuyang and Yuhang. No permission was needed to use fields at these facilities. Authors also confirmed that the study did not involve endangered or protected species.

### Field experiments

The 2010 and 2011 experiments were conducted in two paddy fields with different fertility levels (soil fertility 1 and 2) at the China National Rice Research Institute (CNRRI) (120.2′ E, 30.3′ N, 11 m above sea level), Zhejiang Province, China. The area has a subtropical monsoon climate with an annual mean temperature of 13–20°C (range from 2°C in January to 39°C in July). Mean annual precipitation is 1200–1600 mm, with about 80% rainfall occurring between April and September. Soil at the experimental site is classified as ferric-accumulic stagnic anthrosol [22]. The cropping system of soil fertility 1 was monoculture rice-fallow without rice straw incorporation, and the cropping system of soil fertility 2 was rice-potato (*Solanum tuberosum*) with rice and potato straw incorporation. After 10 years of continuous farming, the soil properties in the two fields differed and this was noted at the start of the experiment in 2010. The soil in field 1 had 2.46 g total N kg^−1^, 0.53 g total P kg^−1^, 0.25 g available K kg^−1^, 10.73 C mol kg^−1^ cation exchange capacity (CEC), and pH 5.78 (soil fertility 1). The corresponding soil properties in field 2 were 2.96 g total N kg^−1^, 0.72 g total P kg^−1^, 0.31 g available K kg^−1^, 10.80 C mol kg^−1^ CEC, and pH 5.15 (soil fertility 2). The soil test was based on samples taken from the upper 10 cm of the soil. Soil chemical properties at the 10–20 cm depth were also different [23].

Pregerminated seeds of a high-yielding hybrid rice variety, Nei2you6, were hill-seeded manually in the two fields with different fertility levels using five spacing schemes (between and within rows: 25 cm×15 cm, 25 cm×17 cm, 25 cm×19 cm, 25 cm×21 cm, and 25 cm×23 cm). Each hill was planted with three to five seeds. The experiment was replicated four times and subplot size was 30 m^2^. In both years, the fields were puddled in ponded conditions and drained 2 d before seeding. Pre-germinated seeds were sown on the surface of puddled soil on June 2, 2010 and May 30, 2011. Before sowing, seeds were soaked for 24 h and incubated for 24 h for proper germination, and seeds dressed with chemical pesticides to avoid the attack of soil insects, birds, and rodents.

To the crop, 225 kg N ha^−1^ (as urea) was applied in three splits in a ratio of 5∶3∶2 at basal, tillering, and earing stage, respectively. Potassium at 180 kg K_2_O ha^−1^ was applied in two splits: 50% as basal dressing and 50% as topdressing at earing in the form of KCl. Phosphorus at 75 kg P_2_O_5_ ha^−1^ in the form of calcium superphosphate was applied as basal. Water management followed standard farmers’ practice, in which the field was drained 2 d before seeding. There was no standing water on the soil surface from sowing to the three-leaf stage of rice, and then the field was reflooded and a water depth of 1 to 3 cm was maintained until the end of the tillering period. After this, water was drained for 7–10 days to control unproductive tillers and later, a water depth of 3–5 cm was maintained until the grain-filling stage. Weed and insects were intensively controlled by chemicals to avoid biomass and yield loss.

In 2010, the number of tillers from 30 hills of rice was counted at 1-week intervals, starting from 20 d after sowing (DAS) to panicle initiation stage. Tillers with at least one visible leaf were included. In 2011, 30 hills of rice plants were sampled at heading and maturity to calculate average panicle number per hill. In both years, six representative hills of rice plants from each plot were separately sampled at heading and divided into leaf blades, stem plus sheath, and panicles. Green leaves were grouped into the top three green leaves and others, and their leaf area was measured with a leaf area meter (LI-3000A, LICOR, Lincoln, NE, USA). LAI was calculated as the total green leaf area of the plant, divided by the corresponding ground sampling area. All samples were oven-dried at 80°C until constant weight for determination of aboveground dry weight at flowering. At maturity, 30 hills were sampled diagonally from each subplot, panicle numbers were counted for each hill to determine the number of panicles per m^2^. Six representative hills of rice plants were selected to determine aboveground total dry weight and harvest index. Grain yield per hill was measured from the sampled 30 hills and grain yield per shoot was calculated as the ratio of yield per hill to panicle numbers per hill. In each subplot, grain yield was determined from a harvesting area of 30 m^2^ and converted to kg ha^−1^ at 14% moisture content. Panicles were hand-threshed and filled spikelets were separated from unfilled spikelets by submerging them in tap water. Dry weights of straw, rachis, and filled and unfilled spikelets were determined after oven drying at 80°C to constant weight. Three subsamples of 20 g of filled spikelets were taken and counted to determine grain weight (20 divided by the counted filled spikelets number); and the number of filled spikelets was calculated as total dry weight of filled spikelets divided by grain weight. All unfilled spikelets were counted and the number of total spikelets (filled and unfilled) was calculated. Aboveground total dry weight at maturity included the total dry matter of straw, rachis, and filled and unfilled spikelets. Dry weight accumulation during the grain-filling phase was calculated as the difference in total aboveground dry weight between flowering and maturity stages. Spikelets per panicle (ratio of total spikelets number to panicles number), grain-filling percentage (ratio of filled spikelets number to total spikelets number), and harvest index (ratio of dry grain yield to total aboveground biomass dry weight) were calculated.

### On-farm demonstration experiments

On-farm demonstrations were conducted in 2012 and 2013 in Fuyang (120.0′ E, 30.1′ N; 14 m above sea level) and Yuhang (120.3′ E, 30.4′ N; 40 m above sea level), Zhejiang Province. Soil at both sites is classified as ferric-accumulic stagnic anthrosol [22]. The soil properties in Fuyang were 2.48 g total N kg-1, 0.56 g total P kg-1, 0.26 g available K kg-1, and pH 5.63. The soil properties in Yuhang were 2.66 g total N kg-1, 0.62 g total P kg-1, 0.32 g available K kg-1, and pH 5.75. The hybrid rice variety used in Fuyang was Nei2you6. In Yuhang, high-yielding hybrid rice varieties Yongyou9 and Yongyou12 were grown. These high-yielding hybrid rice varieties are used widely in south China under a single rice cropping system. Three treatments were included at each site: mechanical hill seeding, broadcasting, and transplanting. Hill seeding was done by using a newly developed hill-seeder ZB-10 (Wang et al 2010) at a spacing of 25 cm×17 cm, three to five seeds per hill, and the seed rate was 21.0 to 25.5 kg ha^−1^. This spacing was chosen because the highest grain yield was noted in this treatment in the 2010 and 2011 experiments. Broadcasting and transplanting were standard farmers’ practices. In the broadcasting treatment, sprouted seeds were broadcast at a seeding rate of 33.8 kg ha^−1^. In the transplanting treatment, seedlings with four to five leaves were transplanted at a spacing of 30 cm×20 cm; two to three seedlings per hill. In the mechanical hill seeding and broadcasting treatments, seed treatment was the same as used in field experiments in 2010 and 2011; all varieties were seeded on the same day (June 2) in both years. Because direct seeding could facilitate timely establishment of rice and earlier crop maturity by 7–10 d, the nursery for the transplanting treatment was raised on May 25 and transplanted on June 18 in both years. The planting area of each treatment was 3000 m^2^, a total of 9000 m^2^ for each variety. Fertilizer and water management in the three treatments were the same as that described for the field experiments in 2010 and 2011. Management operations such as weeding, irrigation, and plant protection measures were done as needed.

At maturity stage, a 5-m^2^ area was harvested diagonally from six places in each treatment for yield determination, and grain yield was calculated at 14% moisture content. Ten hills were sampled diagonally from each 5-m^2^ harvest area to determine yield components in the mechanical hill seeding and transplanting treatments, and a 0.5-m^2^ area from each 5-m^2^ harvest area in the broadcasting treatment. Panicles were hand-threshed and filled and unfilled grains were separated. Dry weights of filled and unfilled spikelets were determined after oven drying samples at 80°C to constant weight. Three subsamples of 20 g of filled spikelets were taken and counted to determine weight and total number of filled spikelets. Unfilled spikelets were counted and grain-filling percentage was calculated.

### Statistical analyses

In the field experiments, normal distribution of data was tested for grain yield, yield components, productive tiller percentage, aboveground dry weight, and harvest index by Q-Q plots using SPSS 11.5 software, and no data transformation was needed to meet the assumptions of normality and constant variance. Data were analyzed following analysis of variance (ANOVA), and differences among treatments were compared based on the least significant difference (LSD) test at the 5% level of probability. The statistical model used in the analysis of grain yield per hectare, per hill, and per shoot included the sources of variation due to replication, seeding density, soil fertility, year, and the interactions of seeding density×soil fertility, seeding density×year, soil fertility×year, and seeding density×soil fertility×year. The bivariate correlations between grain yield and yield components, including grain weight, panicles per hectare and per hill, spikelets per panicle, and grain-filling percentage, were conducted using the Pearson correlation coefficient (two-tailed).

In the on-farm demonstration experiments, ANOVA and mean comparisons were based on the LSD test at the 0.05 probability level for each variety and year (SPSS 11.5).

## Results

### Field experiments

In both years, a significant grain yield response to seeding density was observed (Table 1). Grain yield per hill increased consistently with decreases in seeding density. The plants grown at 25 cm× 23 cm spacing had the highest grain yield per hill and plants grown at 25 cm×15 cm spacing had the lowest. The rate of change in grain yield per hectare with seeding spacing, however, was different between soil fertility 1 and 2. In soil fertility 1, 25 cm×17 cm spacing had the highest grain yield per ha, while 25 cm×15 cm spacing had the lowest value. In soil fertility 2, highest grain yield per ha was obtained at 25 cm×17 cm spacing, but, beyond this, grain yield decreased with an increase in seeding spacing and rice plants grown at 25 cm× 23 cm produced the lowest grain yield per ha. There were large differences in grain yield per shoot among the five seeding spacings in both soil fertility fields. In soil fertility 1, the spacing of 25 cm× 23 cm produced the maximum grain yield per shoot and the lowest value was observed at 25 cm×15 cm spacing. In soil fertility 2, the response of grain yield per shoot to seeding density was the same as that for grain yield per ha; 25 cm×17 cm had the maximum and 25 cm× 23 cm had the minimum grain yield per shoot. The difference in grain yield between the two years was nonsignificant (Table 1).

**Table 1:** Grain yield of a high-yielding hybrid rice variety Nei2you6 grown at five seeding spacings in soil fertility 1 and soil fertility 2 at the China National Rice Research Institute, Zhejiang Province, China, in 2010 and 2011.

Year	Field	Seeding spacing	Grain yield (kg ha^−1^)	Grain yield (g hill^−1^)	Grain yield (g shoot^−1^)
2010	Soil fertility 1	25 cm×15 cm	7804 c	29.37 d	3.09 d
		25 cm×17 cm	8348 a	36.01 c	3.49 b
		25 cm×19 cm	8152 ab	38.05 bc	3.41 bc
		25 cm×21 cm	7892 bc	41.65 b	3.36 c
		25 cm×23 cm	7938 b	46.05 a	3.68 a
	Soil fertility 2	25 cm×15 cm	9133 b	34.69 d	3.64 b
		25 cm×17 cm	9433 a	40.63 c	3.84 a
		25 cm×19 cm	8801 c	42.08 bc	3.68 b
		25 cm×21 cm	8437 d	45.09 b	3.50 c
		25 cm×23 cm	8311 d	48.35 a	3.38 d
2011	Soil fertility 1	25 cm×15 cm	7842 c	29.79 e	3.14 d
		25 cm×17 cm	8573 a	36.80 d	3.56 b
		25 cm×19 cm	8218 b	39.61 c	3.55 b
		25 cm×21 cm	8121 b	43.24 b	3.48 c
		25 cm×23 cm	8140 b	47.19 a	3.70 a
	Soil fertility 2	25 cm×15 cm	9380 b	35.39 d	3.71 bc
		25 cm×17 cm	9611 a	41.13 c	3.88 a
		25 cm×19 cm	8942 c	42.91 bc	3.76 b
		25 cm×21 cm	8831 c	47.14 ab	3.66 c
		25 cm×23 cm	8582 d	49.94 a	3.49 d
Analysis of variance					
Seeding density (A)			*	*	*
Soil fertility (B)			*	*	*
Year (C)			NS	NS	NS
A×B			*	*	*
A×C			NS	NS	NS
B×C			NS	NS	NS
A×B×C			*	*	*

Within a column for each year, means followed by the same letters are not significantly different according to the LSD test (0.05).

*Significance at the 0.05 level based on analysis of variance.

NS denotes nonsignificance based on analysis of variance.

In both years, there was no significant difference in grain weight among different spacing treatments in both fields (Table 2). The number of panicles per hill consistently increased with increases in seeding spacing in both years and both fields. Across years, with increases in seeding spacing from 25 cm×15 cm to 25 cm×23 cm, panicles per hill increased by 34.2% in soil fetility 1 and by 50.0% in soil fertility 2. In both years, the number of panicles per m^2^ was not significantly different at spacing ranging from 25 cm×17 cm to 25 cm× 21 cm in soil fertility 1; the maximum and minimum number of panicles per m^2^ was observed at 25 cm×15 cm and 25 cm× 23 cm spacing, respectively. In soil fertility 2, 25 cm×15 cm spacing had higher number of panicles per m^2^ in 2010, while no difference was observed among other treatments in this year and among all spacings in 2011. In both years, 25 cm×15 cm spacing had the minimum number of spikelets per panicle in soil fertility 1, while no difference was found among treatments in soil fertility 2. Grain-filling percentage in 2011 was higher than that in 2010 in both soil fertility fields. The plants grown at 25 cm× 23 cm spacing had the highest and 25 cm×15 cm spacing had the lowest grain-filling percentage in soil fertility 1. Meanwhile, in soil fertility 2, 25 cm× 17 cm spacing had the highest grain filling percentage and 25 cm× 23 cm spacing had the lowest value in both years.

**Table 2:** Yield components of a high-yielding hybrid rice variety Nei2you6 grown at five seeding spacings in soil fertility 1 and soil fertility 2, China National Rice Research Institute, Zhejiang Province, China, 2010 and 2011.

Year	Field	Seeding spacing	Spikelets panicle^−1^ (no.)	Grain filling (%)	Grain weight (mg)	Panicles m^−2^ (no.)	Panicles hill^−1^ (no.)
2010	Soil fertility 1	25 cm×15 cm	115.52 b	76.82 e	30.30 a	240.97 a	9.03 c
		25 cm×17 cm	125.92 a	77.74 d	30.24 a	228.08 b	9.71 bc
		25 cm×19 cm	126.70 a	78.85 c	30.46 a	223.75 b	10.60 b
		25 cm×21 cm	125.42 a	79.99 b	30.72 a	226.41 b	11.91 a
		25 cm×23 cm	127.55 a	81.51 a	30.52 a	210.76 c	12.11 a
	Soil fertility 2	25 cm×15 cm	135.01 a	81.63 b	30.73 a	247.01 a	9.25 cd
		25 cm×17 cm	135.78 a	83.15 a	30.44 a	238.86 b	10.17 c
		25 cm×19 cm	130.83 a	81.44 b	30.22 a	229.02 b	10.86 c
		25 cm×21 cm	135.80 a	78.06 c	30.56 a	234.84 b	12.36 b
		25 cm×23 cm	133.09 a	74.11 d	30.33 a	236.59 b	13.59 a
2011	Soil fertility 1	25 cm×15 cm	127.68 b	84.90 c	33.48 a	266.33 a	9.98 c
		25 cm×17 cm	142.00 a	86.66 b	34.10 a	257.20 ab	10.95 bc
		25 cm×19 cm	140.04 a	87.15 b	33.66 a	247.31 b	11.72 b
		25 cm×21 cm	135.88 a	86.65 b	33.28 a	245.27 b	12.91 a
		25 cm×23 cm	140.97 a	90.09 a	33.74 a	232.94 c	13.39 a
	Soil fertility 2	25 cm×15 cm	143.37 a	86.67 b	32.63 a	262.29 a	9.83 e
		25 cm×17 cm	147.10 a	90.07 a	32.98 a	258.76 a	11.01 d
		25 cm×19 cm	144.61 a	90.02 a	33.40 a	253.12 a	12.00 c
		25 cm×21 cm	147.12 a	84.56 c	33.10 a	254.42 a	13.40 b
		25 cm×23 cm	147.09 a	81.91 d	33.53 a	261.49 a	15.03 a

Within a column for each site, means followed by the same letters are not significantly different according to the LSD test (0.05).

**Figure 1:**
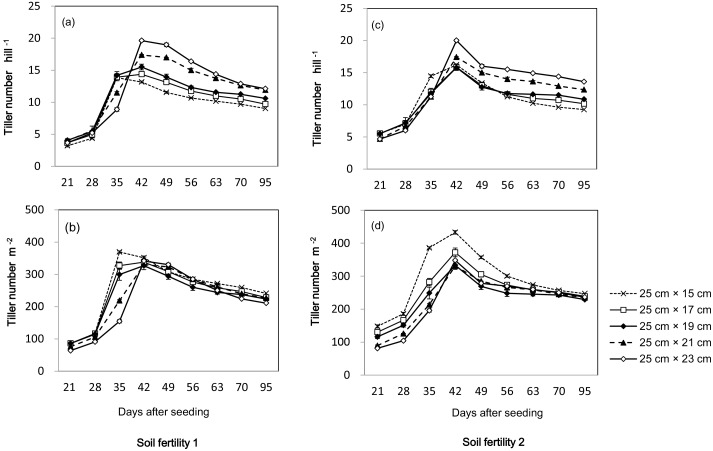
Tillers per hill (a, c) and tillers per m^2^ (b, d) of a high-yielding hybrid rice variety Nei2you6 grown at five plant spacings in soil fertility 1 (a, b) and soil fertility 2 (c, d). China National Rice Research Institute, Zhejiang Province, China, 2010.

In 2010, maximum tiller number per hill was observed at 25 cm×15 cm spacing at 35 DAS in soil fertility 1, while at 42 DAS at other seeding spacings in soil fertility 1 and all treatments in soil fertility 2 (Figure 1). A decreasing trend of tiller number per hill was observed after the reproductive stage, irrespective of treatment. Maximum tiller numbers per hill and per m^2^ were affected by seeding density. In both soil fertility fields, maximum tiller number per hill increased with increases in seeding spacing; 25 cm× 23 cm spacing had the highest tiller number per hill and 25 cm×15 cm spacing had the lowest. However, maximum tiller number per m^2^ in both fields decreased with increases in seeding spacing; it was highest at 25 cm×15 cm spacing. From the maximum tillering to flowering stage, the difference in tiller number per m^2^ among treatments gradually decreased with the reduction in unproductive tillers.

**Figure 2:**
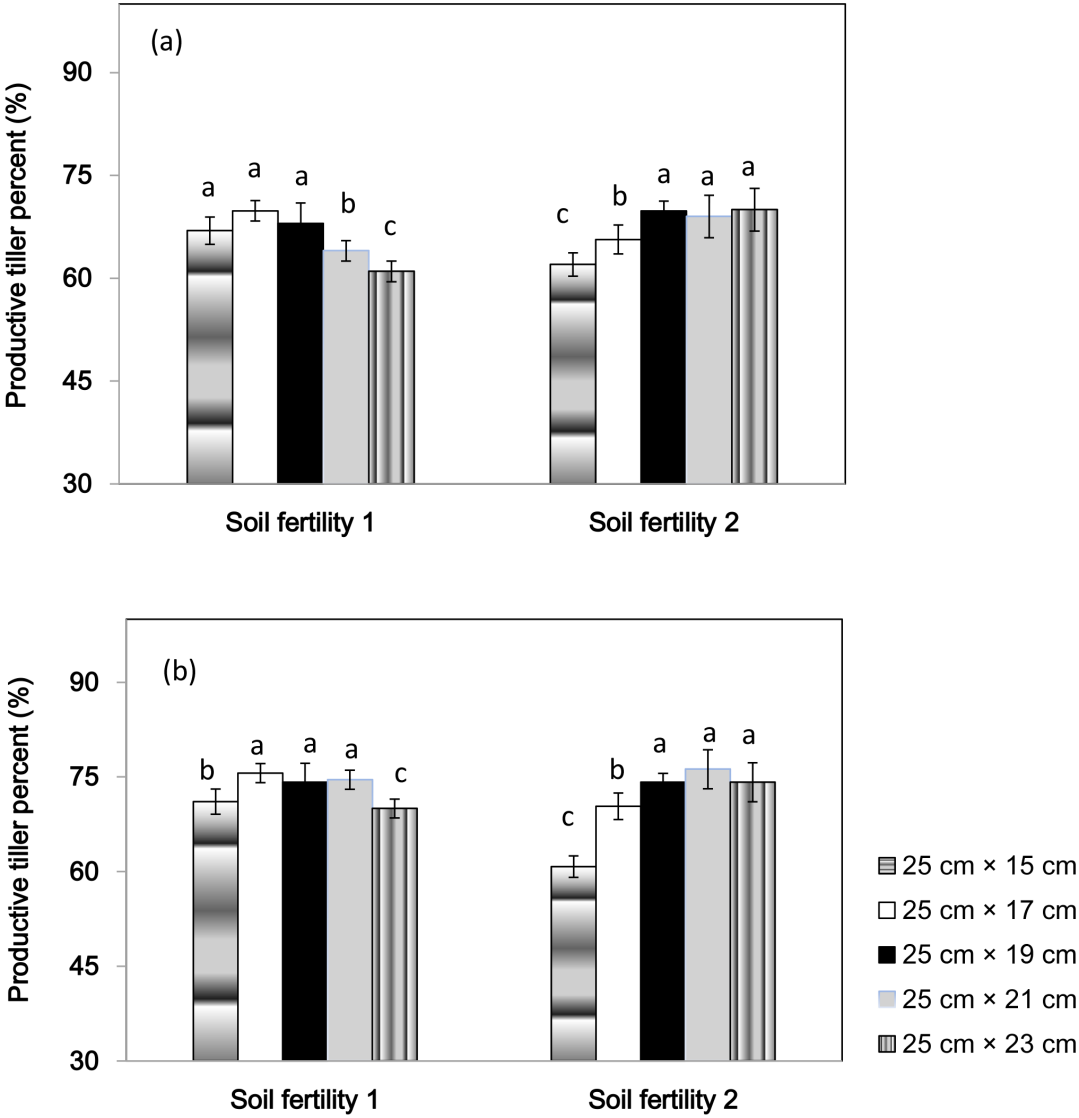
Productive tiller percentage of a high-yielding hybrid rice variety Nei2you6 grown at five seeding spacings in soil fertility 1 and soil fertility 2. China National Rice Research Institute, Zhejiang Province, China, 2010 (a) and 2011 (b).

The effects of seeding density on the percentage of productive tillers were also different between the two soil fertility fields (Figure 2). In soil fertility 1, 25 cm× 23 cm spacing had the lowest productive tiller percentage in both years, and no significant difference was observed among spacings ranging from 25 cm×15 cm to 25 cm×19 cm in 2010 and from 25 cm×17 cm to 25 cm×21 cm in 2011. In both years, in soil fertility 2, 25 cm×15 cm spacing had the lowest productive tiller percent, followed by 25 cm×17 cm spacing, and both these spacings had significantly lower productive tillers than the other ones.

Although leaf area per hill at flowering consistently increased in both years with increases in seeding spacing in both soil fertility fields, the change in leaf area per shoot was different between the two fields (Table 3). In both years, total leaf area per shoot and top three leaf blades per shoot increased with increases in seeding spacing in soil fertility 1, while they decreased in soil fertility 2. The highest leaf area index (LAI) in soil fertility 1 was obtained at 25 cm×15 cm spacing, followed by that at 25 cm×23 cm spacing. The highest LAI in soil fertility 2 was also observed at 25 cm×15 cm spacing, while it gradually decreased with increases in seeding spacing; 25 cm×23 cm spacing had the lowest LAI in both years.

**Table 3:** Leaf area index (LAI), leaf area per hill, and leaf area per shoot at flowering of a high-yielding hybrid rice variety Nei2you6 grown at five seeding spacings in soil fertility 1 and soil fertility 2, China National Rice Research Institute, Zhejiang Province, China, 2010 and 2011.

Year	Field	Seeding spacing	LAI	Leaf area (m^2^ per hill)			Leaf area (×10^−3^ m^2^ per shoot)		
				Total	Top 3 leaves	Others	Total	Top 3 leaves	Others
2010	Soil fertility 1	25 cm×15 cm	5.72 a	0.21 e	0.12 d	0.11 c	26.87 c	14.16 c	12.71 b
		25 cm×17 cm	5.39 c	0.23 d	0.13 d	0.11 c	27.76 c	15.18 c	12.58 b
		25 cm×19 cm	5.42 bc	0.26 c	0.14 c	0.12 b	28.78 b	16.15 b	12.63 b
		25 cm×21 cm	5.49 b	0.29 b	0.16 b	0.13 a	29.08 b	16.46 b	12.62 b
		25 cm×23 cm	5.53 b	0.32 a	0.18 a	0.13 a	31.18 a	17.80 a	13.35 a
	Soil fertility 2	25 cm×15 cm	7.03 a	0.27 e	0.16 c	0.10 d	35.66 a	21.02 a	13.64 c
		25 cm×17 cm	7.07 a	0.30 d	0.17 bc	0.13 c	35.55 a	20.97 a	14.57 b
		25 cm×19 cm	6.68 b	0.32 c	0.18 b	0.13 c	34.67 b	19.59 b	15.07 b
		25 cm×21 cm	6.66 b	0.35 b	0.18 b	0.16 b	34.06 c	17.89 c	16.17 a
		25 cm×23 cm	6.46 c	0.37 a	0.19 a	0.17 a	32.43 d	17.05 c	15.37 b
2011	Soil fertility 1	25 cm×15 cm	6.20 a	0.23 d	0.12 d	0.11 d	29.11 c	15.34 c	13.77 ab
		25 cm×17 cm	5.61 d	0.23 d	0.13 d	0.11 d	28.90 c	15.80 c	13.10 b
		25 cm×19 cm	5.88 c	0.28 c	0.16 c	0.12 c	31.18 b	17.49 b	13.69 ab
		25 cm×21 cm	5.83 c	0.31 b	0.18 b	0.13 b	31.88 b	17.48 b	13.40 b
		25 cm×23 cm	5.99 b	0.34 a	0.20 a	0.15 a	33.78 a	19.28 a	14.47 a
	Soil fertility 2	25 cm×15 cm	7.77 a	0.29 e	0.18 c	0.12 e	38.30 a	23.24 a	15.08 c
		25 cm×17 cm	7.51 b	0.32 d	0.19 b	0.13 d	37.75 a	22.27 ab	15.47 c
		25 cm×19 cm	7.24 c	0.34 c	0.20 ab	0.15 c	37.55 a	21.23 b	16.33 b
		25 cm×21 cm	7.01 d	0.37 b	0.19 b	0.17 b	35.81 b	18.81 c	17.00 a
		25 cm×23 cm	6.86 d	0.39 a	0.21 a	0.19 a	34.43 b	18.11 c	16.33 b

Green leaves per hill were subdivided into top three green leaves and others. Leaf area per shoot was calculated as the ratio of leaf area per hill to productive tiller number per hill.

Within a column for each site, means followed by the same letters are not significantly different according to the LSD test (0.05).

**Figure 3:**
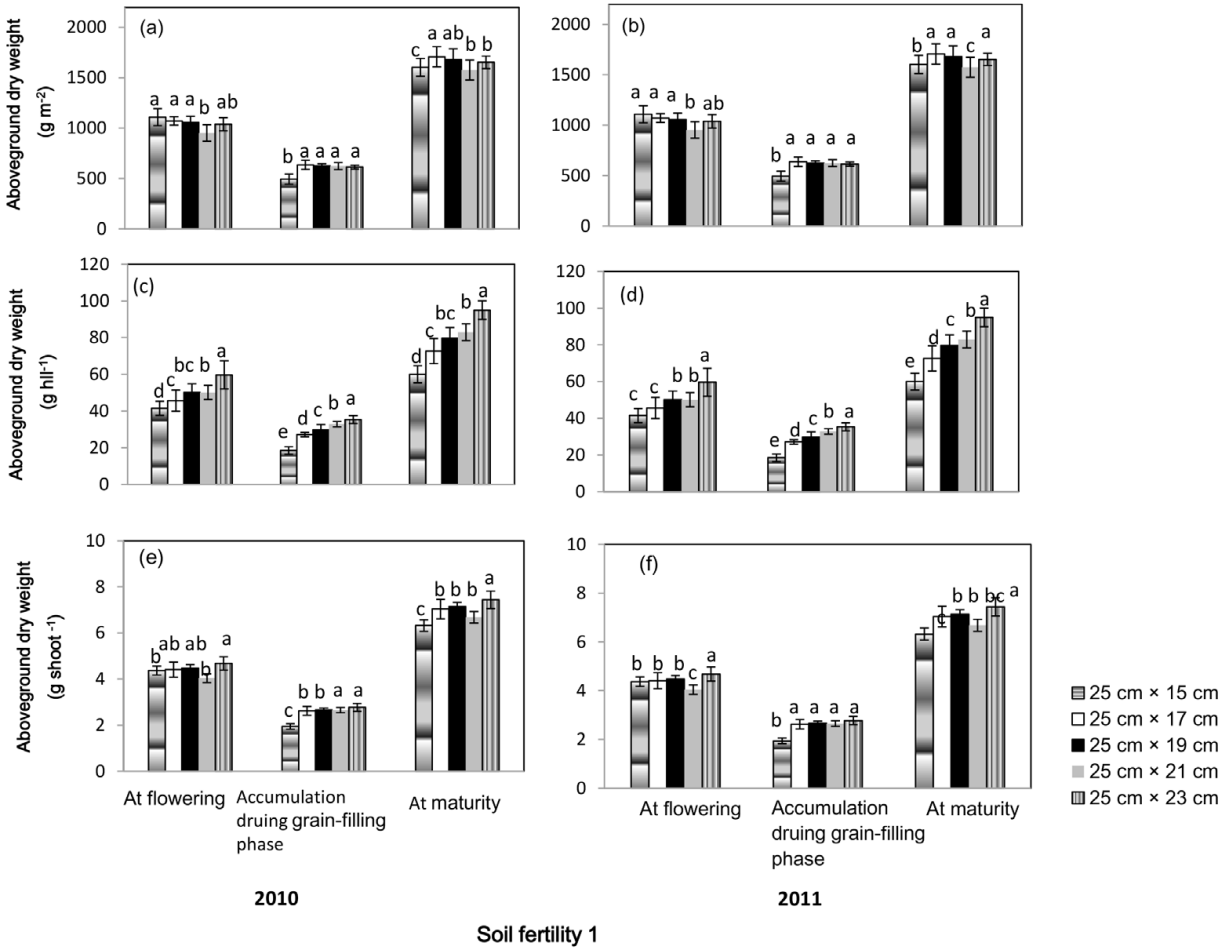
Aboveground dry weight at flowering and maturity, and dry weight accumulation during grain-filling phase of a high-yielding hybrid rice variety Nei2you6 grown at five seeding spacings in soil fertility 1 at the China National Rice Research Institute, Zhejiang Province, China, in 2010 and 2011. Aboveground dry weight was calculated as dry weight per m^2^ (a, b), dry weight per hill (c, d), and dry weight per shoot (e, f). Dry weight per shoot was the ratio of dry weight per hill to productive tiller number per hill.

**Figure 4:**
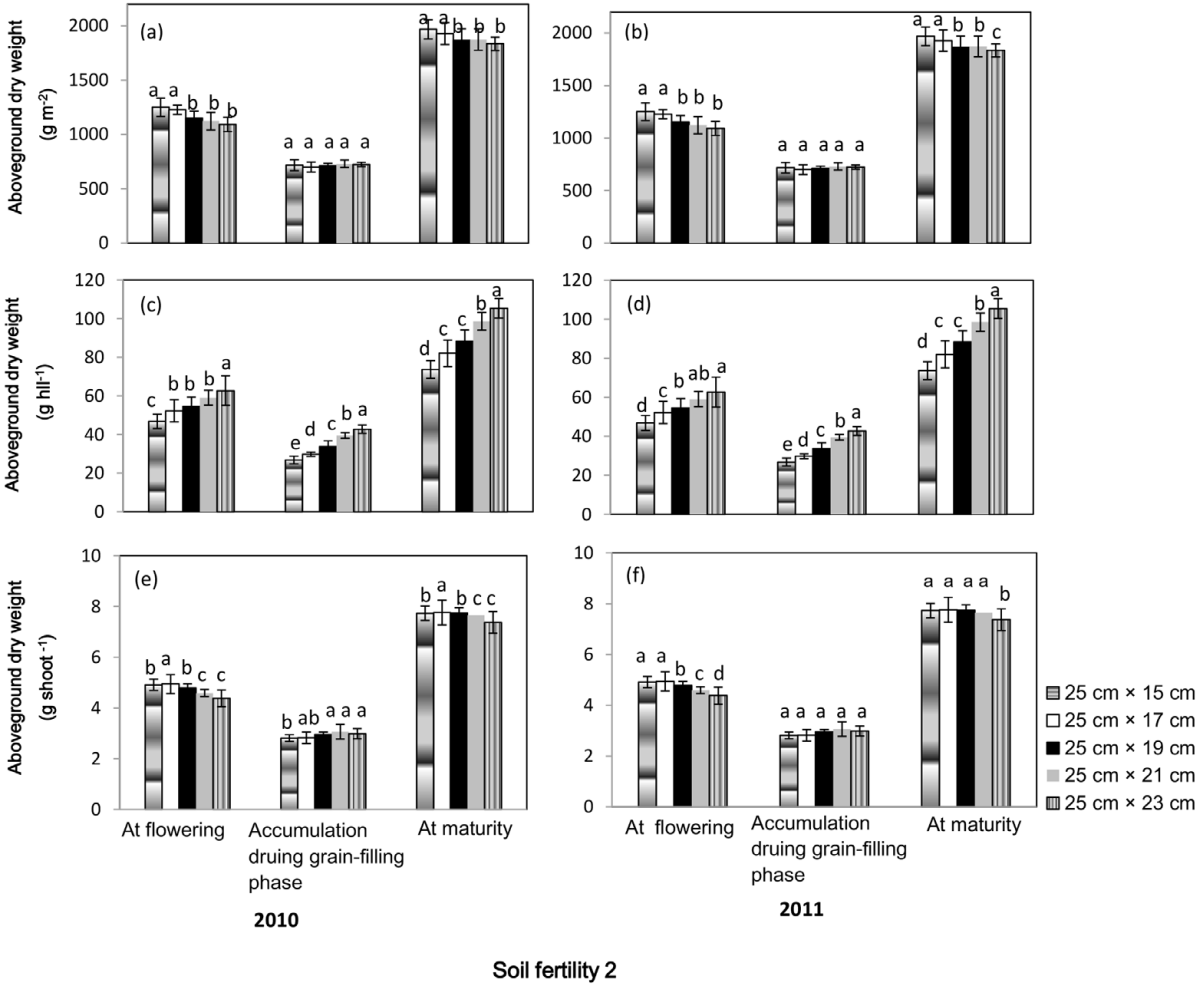
Aboveground dry weight at flowering and maturity, and dry weight accumulation during grain-filling phase of a high-yielding hybrid rice variety Nei2you6 grown at five seeding spacings in soil fertility 2 at the China National Rice Research Institute, Zhejiang Province, China, in 2010 and 2011. Aboveground dry weight was calculated as dry weight per m^2^ (a, b), dry weight per hill (c, d), and dry weight per shoot (e, f). Dry weight per shoot was the ratio of dry weight per hill to productive tiller number per hill.

Aboveground dry weight per hill at flowering and maturity, and dry weight accumulation per hill during grain-filling phase increased with increases in seeding spacing; 25 cm× 23 cm spacing had the highest values and 25 cm×15 cm spacing had the lowest in both soil fertility fields in both years (Figures 3 and 4). In terms of aboveground dry weight per shoot, a difference existed between the two soil fertility fields. In both years, the highest aboveground dry weight per shoot at flowering and maturity was observed at 25 cm× 23 cm spacing in soil fertility 1; rice plants grown at 25 cm×15 cm spacing had the lowest dry weight accumulation per shoot during the grain-filling phase and aboveground dry weight per shoot at maturity. In soil fertility 2, no significant difference in dry weight accumulation during the grain-filling phase was observed in spacing ranging from 25 cm×17 cm to 25 cm× 23 cm in both years; and aboveground dry weight per shoot at flowering and maturity decreased with increases in spacing from 25 cm×17 cm to 25 cm× 23 cm. Significant differences also existed in aboveground dry weight per m^2^ between the two soil fertility fields. In soil fertility 1, no significant difference was found for aboveground dry weight per m^2^ at flowering at spacings ranging from 25 cm×15 cm to 25 cm×19 cm. Rice plants grown at 25 cm×15 cm spacing had the lowest dry weight accumulation during grain filling, and no significant difference was observed at spacings ranging from 25 cm×17 cm to 25 cm×23 cm. Aboveground dry weight at maturity was highest at 25 cm×17 cm spacing in both years and lowest at 25 cm×15 cm spacing in 2010 and at 25 cm×15 cm spacing in 2011. In soil fertility 2, no significant difference in dry weight accumulation during grain-filling phase was observed among the treatments; aboveground dry weights per m^2^ at flowering and maturity were significantly higher at 25 cm×15 cm and 25 cm×17 cm spacing than at spacing wider than 25 cm×19 cm.

**Figure 5:**
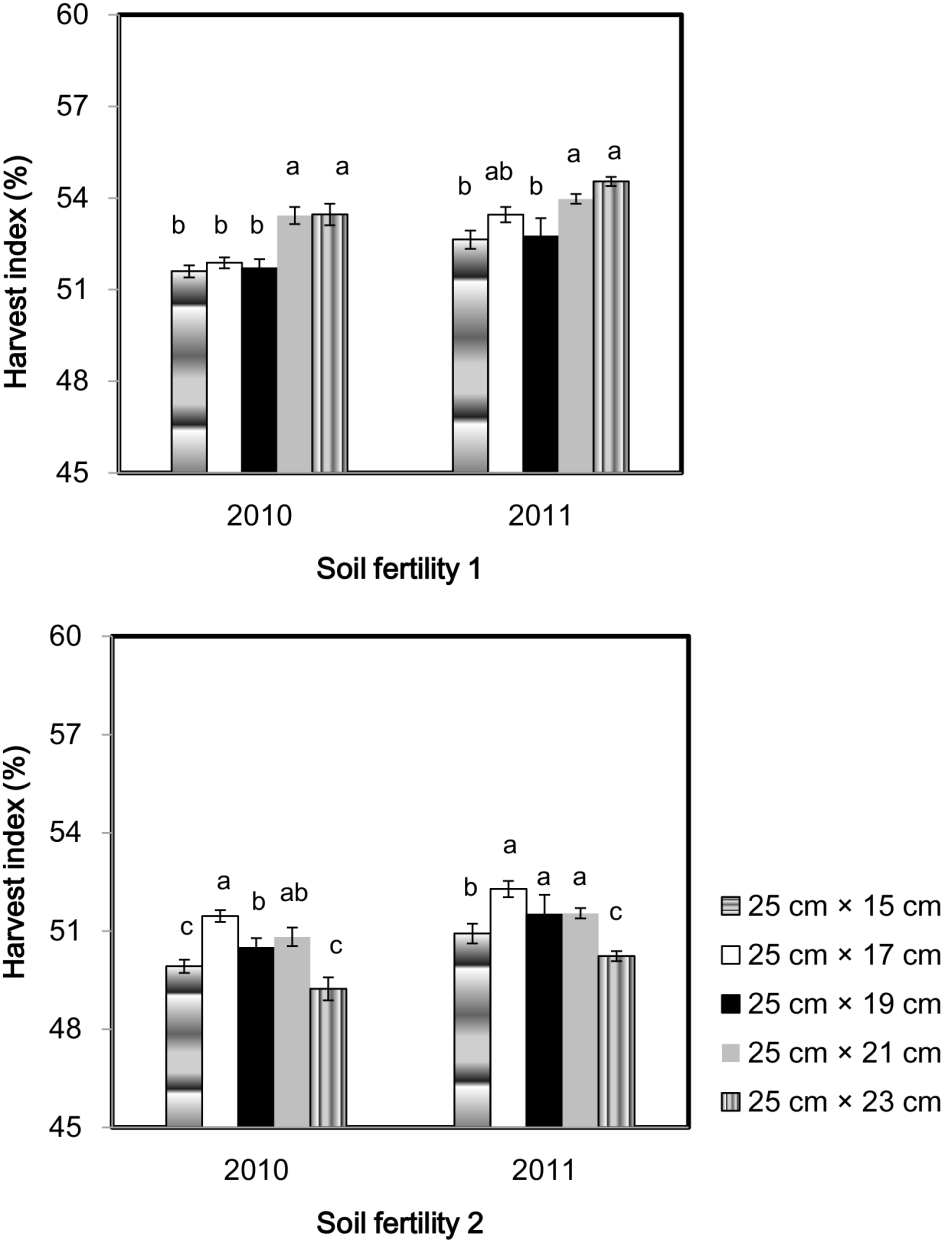
Harvest index of a high-yielding hybrid rice variety Nei2you6 grown at five seeding spacings in soil fertility 1 and soil fertility 2 at the China National Rice Research Institute, Zhejiang Province, China, in 2010 and 2011. Dry weight per shoot was calculated as the ratio of dry weight per hill to productive tiller number per hill.

The effect of seeding density on harvest index (HI) was also different between the two soil fertility fields (Figure 5). In soil fertility 1, 25 cm×23 cm spacing and 25 cm×21 cm spacing had higher HI than that at the other three narrower spacings in both years. In soil fertility 2, plants grown at spacings ranging from 25 cm×17 cm to 25 cm×21 cm had significantly higher HI than plants grown at 25 cm×15 cm and 25 cm×15 cm spacings.

### On-farm demonstration experiments

**Figure 6:**
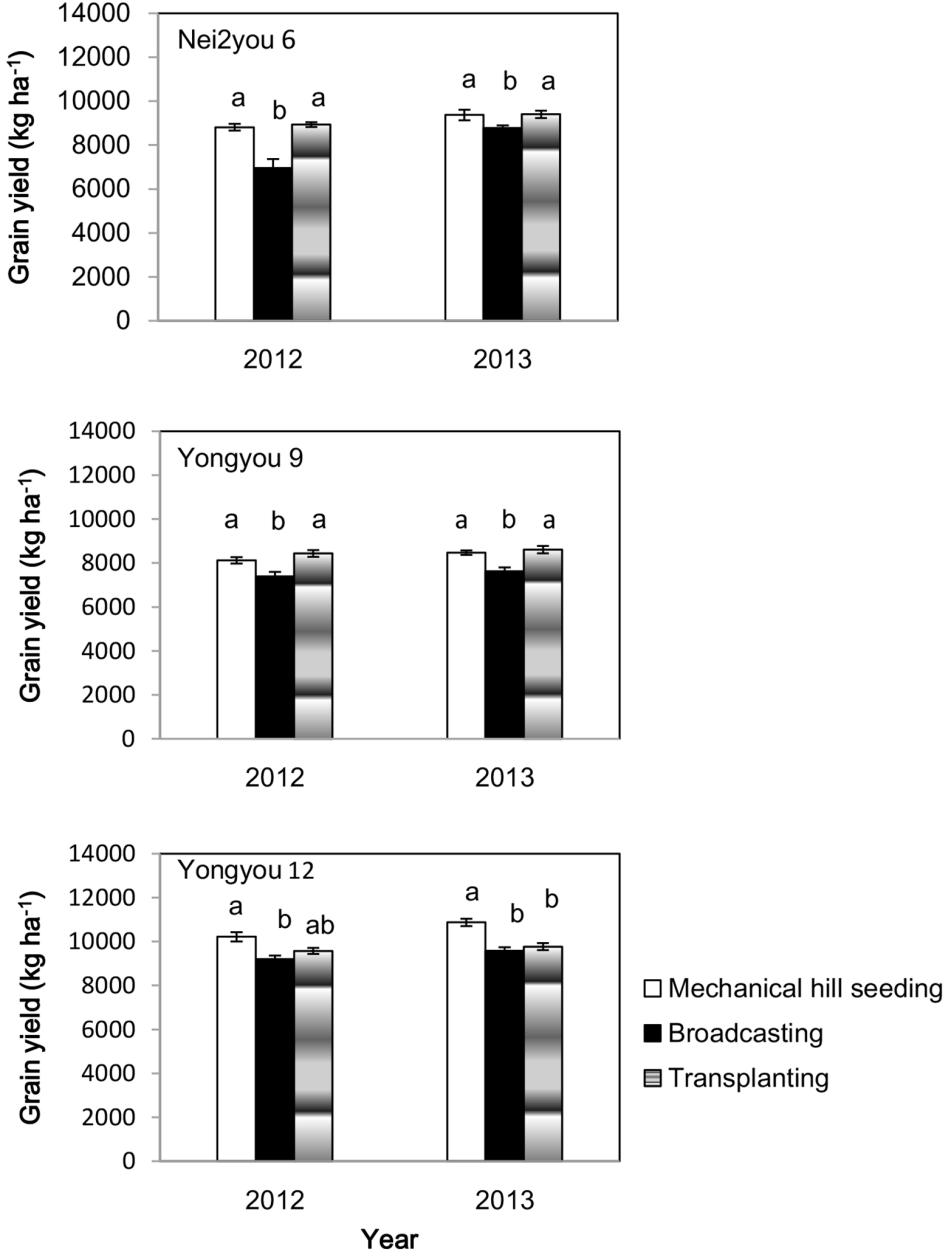
Grain yield of three high-yielding hybrid rice varieties under three plant establishment methods in Fuyang and Yuhang, Zhejiang Province, in 2012 and 2013. Plant spacing for mechanical hill seeding was 25 cm×17 cm; broadcasting and transplanting done according to standard farmer’s practice.

In the on-farm demonstration experiments in 2012 and 2013, mechanical hill seeding, using seeder ZB-10 at a spacing of 25 cm×17 cm, outperformed broadcasting in terms of grain yield for all three high-yielding hybrid rice varieties, increasing grain yield by 10.4% in Yongyou 9 and 12.3% in Yonyou 12 across the 2 years. In 2012, variety Nei2you6 produced 26.6% higher grain yield in mechanical hill seeding compared with broadcasting because more than 30% of the plants lodged in the broadcast culture due to heavy rain during the later grain-filling phase (Figure 6). In both years, no significant difference in grain yield between mechanical hill-seeded rice and transplanted rice was observed for Nei2you 6 and Yonyou 9. Yongyou 12 produced more than 1000 kg ha^−1^ grain yield in the mechanical hill seeding treatment across 2 years, increasing grain yield by 9.1% compared with that of transplanted rice.

Mechanical hill-seeded rice had more spikelets per panicle and fewer panicles per m^2^ compared with broadcasting, but fewer spikelets per panicle and more panicles per unit area than transplanted rice (Table 4). Across the years, mechanical hill-seeded hybrid rice varieties Nei2you 6, Yongyou 9, and Yonyou 12 had 36.0%, 28.3%, and 33.1% higher number of spikelets per panicle, and 17.4%, 16.7%, and 14.3% lower number of panicles per m^2^, respectively, compared with the broadcast culture. Compared with the transplanted treatment, 7.3%, 15.5%, and 13.4% decline in spikelets per panicle, and 11.2%, 20.7%, and 28.5% increase in panicle number per m^2^ were observed in mechanical hill-seeded hybrid rice varieties Nei2you 6, Yongyou 9, and Yonyou 12, respectively.

**Table 4:** Grain yield components of three high-yielding hybrid varieties under three plant establishment methods in Fuyang and Yuhang, Zhejiang Province, China, in 2012 and 2013.

Variety	Year	Treatment	Spikelets panicle^−1^ (no.)	Grain filling (%)	Grain weight (mg)	Panicles m^−2^ (no.)
Nei2you 6	2012	Mechanical hill seeding	176.03 b	83.25 a	26.76 b	235.56 b
		Broadcasting	130.82 c	80.32 b	26.72 b	283.65 a
		Transplanting	193.86 a	84.41 a	27.18 a	213.09 c
	2013	Mechanical hill seeding	174.03 b	85.75 a	27.42 a	250.60 b
		Broadcasting	126.57 c	82.40 b	26.84 b	305.00 a
		Transplanting	183.58 a	86.52 a	27.41 a	224.30 b
Yongyou 9	2012	Mechanical hill seeding	169.87 b	85.16 b	21.88 a	251.52 b
		Broadcasting	133.13 c	83.37 c	21.77 a	303.61 a
		Transplanting	203.08 a	87.63 a	22.17 a	210.60 c
	2013	Mechanical hill seeding	166.79 b	87.55 ab	22.79 a	262.00 b
		Broadcasting	129.37 c	85.49 b	22.45 a	313.00 a
		Transplanting	195.33 a	89.61 a	22.62 a	214.90 c
Yongyou 12	2012	Mechanical hill seeding	388.02 b	82.16 a	19.46 b	171.93 b
		Broadcasting	294.69 c	80.24 b	19.79 ab	202.85 a
		Transplanting	457.15 a	84.45 a	20.21 a	136.71 c
	2013	Mechanical hill seeding	389.09 b	84.46 ab	20.71 a	182.90 b
		Broadcasting	289.35 c	82.40 b	20.62 a	211.30 a
		Transplanting	439.71 a	86.52 a	20.62 a	139.50 c

Plant spacing for mechanical hill seeding was 25 cm×17 cm; broadcasting and transplanting done according to standard farmer’s practice.

## Discussion

Our study showed that high-yielding hybrid rice variety Nei2you6 produced maximum grain yield at a seeding spacing of 25 cm×17 cm in two soil fertility fields in both 2011 and 2012. Optimal hill seeding density for hybrid rice has also been reported in previous studies: Peilianyou 986 had the highest grain yield at a spacing of 25 cm×18 cm [20]; Jinyou 207 had the highest grain yield when hill-seeded at 30 cm×20 cm spacing [17]; and hill-seeded Peizataifen and Yuyouxiangzhang [19], [ 20] gave the highest grain yield at 25 cm×14 cm. However, these studies were conducted at one location in a single season and used only one hybrid rice variety with one to three seeding rates. It is thus difficult to conclude which seeding density can fullfill the yield potential of most high-yielding hybrid rice varieties. In our study, we compared plant yield and dry weight at five hill seeding spacings in two fields of different fertilities during a 2-year period. Besides that, three high-yielding hybrid rice varieties were mechanically seeded with a hill seeder at 25 cm×17 cm spacing in the on-farm demonstration experiments and compared with broadcasting and transplanting. Mechanical hill seeding outperformed broadcasting in terms of grain yield for all three high-yielding hybrid rice varieties; they also had grain yield equal to or higher than that of transplanted rice. We conclude that, for the south China region, a hill seeding spacing of 25 cm×17 cm, with three to five seeds per hill, is the appropriate density to be used for single cropping of high-yielding hybrid rice.

There have been extensive studies on the relationships between yield and plant density under nonstressed conditions [24]–[29]. For decades, rice reseachers in China have emphasized the importance of regulating the compensatory and competitive relationships among plants to increase grain yield by modifying planting density. In the 1960s, dense planting to increase panicle number per unit area was recommended in inbred rice production [30], [31]. In the 1980s, with the development of inbred and hybrid rice varieties with large panicles, the advice on rice planting density changed. It was reported that planting at wide spacing decreased plant number per unit area, which improved the morphological traits of the rice plants such as plant height, tiller angle, and leaf angle [32]. It has been widely accepted at present in high-yielding hybrid rice production systems that increasing panicle size, rather than panicle number per unit area, increases yield [32]–[34]. In considering best management practices for rice, the basic concept applied is the use of young seedlings at optimal spacing to stimulate individual plant growth [35]. Recently, on-farm surveys conducted in Zhejiang, China, showed that some farmers transplanted hybrid rice using extremely wide spacing because of rising labor cost. They opined that the reduced plant number caused by low planting density could be compensated for by the improvement in growth of the individual plants [36]. In our study, although grain yield per hill across two years consistently increased with increases in seeding spacing, grain yield per hill of hybrid rice variety Nei2you6 at 25 cm×23 cm spacing was 57.6% and 40.3% higher than at 25 cm×15 cm spacing in soil fertility 1 and 2, respectively. Grain yield per area across two years was highest at 25 cm×17 cm spacing, which was 5.3% and 12.7% higher than at 25 cm×23 cm spacing in soil fertility 1 and 2, respectively. These results suggest that yield per unit area cannot be fully compensated for by the yield increase of individual plants. Therefore, enough seedlings are necessary to achieve high yield. The on-farm surveys in Zhejiang Province also showed that the lowest grain yield was obtained with the widest planting spacing because of the lack of adequate plant population [36].

Rice grain yield per area or per hill is ultimately determined by the number of panicles per area or per hill and the grain yield per panicle. It was reported that yield was positively related to panicle number per unit area [36]–[37]. In our study, there was a significantly positive correlation between yield per hill and panicles per hill (*r* = 0.98**), and a significantly negative correlation between grain yield per ha and panicles per hill (*r* = −0.89*) (Table 5). Panicle number is largely determined by the number of tillers that develop during the vegetative stage [38] and there is a large variation in tillering capacity among rice varieties [39], [40]. Environmental factors and agronomic practices also alter tiller production and survival [41]–[43]. Plant density and fertilization are important factors influencing tiller production in rice [39], [44]–[45]. LAI probably affected tillering by attenuation of light intensity and/or by influencing light quality at the base of the canopy where tiller buds and young tillers are located [46]. Furthermore, there are evidences showing that plant N status and LAI depend on each other in determining tillering [42]. A higher plant N concentration was needed to prevent tillers from dying when LAI was high [42]. In our study, maximum tiller number per hill increased with increases in seeding spacing, suggesting that plant tillering capacity increased with a decrease in seeding density. Compared with soil fertility 1, 17.2% and 10.1% increase in maximun tiller number per hill was obtained at 25 cm×15 cm and 25 cm×17 cm spacing, respectively, in soil fertility 2. However, no difference in maximum tiller number per hill was found between the two soil fertility fields at spacings ranging from 25 cm×19 cm to 25 cm×23 cm, suggesting that the negative effect of dense seeding on plant tillering capacity could be partly compensated for by an increase in nutrition supply in soil fertility 2. However, the rate of change in productive tiller percentage against seeding spacing was different between the two soil fertility fields. The lower productive tiller percentage at 25 cm×15 cm in soil fertility 2 offset its advantage of maximun tiller number per hill, and no significant difference in productive tillers per hill was observed at this spacing between the two fields. A 7% higher productive tillers at 25 cm×23 cm spacing in soil fertility 2 resulted in significantly higher panicle number in this field compared with soil fertility 1. The number of surviving tillers depends mainly on the extent of competition among tillers for carbohydrates produced and N absorbed from the maximum tillering stage to heading [40]. The differences in productive tiller percentage among seeding densities and between two soil fertility fields indicate that nutrition supply could increase panicle number at wide seeding spacing, but it cannot compensate for the negative effect of high seeding density on panicle number per hill because of the reduced productive tiller percentage.

**Table 5:** Correlation coefficients between grain yield and yield components of a high-yielding hybrid rice variety Nei2you6 grown at five seeding spacings at the China National Rice Research Institute, Zhejiang Province, China, in 2010 and 2011.

Yield and yield components	Spikelets per panicle	Grain filling percentage	Grain weight	Panicles per unit area	Panicles per hill	Grain yield per ha	Grain yield per hill
Grain filling percentage	−0.218						
Grain weight	−0.001	−0.802					
Panicles per unit area	0.207	−0.105	−0.449				
Panicles per hill	0.229	−0.842	0.973**	−0.378			
Grain yield per ha	0.159	0.828	−0.947*	0.409	−0.891*		
Grain yield per hill	0.291	−0.729	0.942*	−0.502	0.980**	−0.824	
Grain yield per shoot	0.041	0.965**	−0.791	−0.098	−0.774	0.868	−0.635

*Significant at *p*<0.05.

**Significant at *p*<0.01.

There are compensatory and competitive relationships among tillers and their yield components over a wide range of plant densities [39]. In soil fertility 2, when seeding spacing was increased from 25 cm×17 cm to 25 cm×23 cm, the number of panicles per hill increased by 33.6% in 2010, and by 22.3% in 2011, while grain yield per shoot declined by 12.0% and by 10.0%, respectively. Grain filling percentage declined with increases in seeding spacing in both years in soil fertility 2, and no significant differences in spikelets per panicle and grain weight were found among treatments, suggesting that grain filling percentage decreased with the increase in productive tillers per hill, which resulted in the decline in yield per shoot. However, there was an interaction between seeding density and soil fertility. Less change in grain yield per shoot was in soil fertility 1 than soil fertility 2 at seeding spacings ranging from 25 cm×17 cm to 25 cm×21 cm. Rice plants grown at 25 cm×23 cm spacing had the highest grain yield per shoot in soil fertility 1, but had the the lowest grain yield per shoot in soil fertility 2. It was reported that high-yielding varieties usually have high biomass accumulation during the reproductive phase [47]–[51]. In our study, although aboveground dry weight per hill at flowering and maturity increased with increases in seeding spacing, aboveground dry weight per shoot was different between the two fertility fields. Rice plants seeded at 25 cm×23 cm spacing had the lowest aboveground dry weight per shoot both at flowering and maturity in soil fertility 2, while they had the highest aboveground dry weight per shoot in soil fertility 1. A different response of leaf area per shoot to seeding spacing was one of the factors that influence dry matter accumulation. The top three-leaf area per shoot increased with increases in seeding spacing in soil fertility 1, while it decreased in soil fertility 2. Considering that there are more panicles per hill in soil fertility 2 than in soil fertility 1 at wider seeding spacing and no extra fertilizer application, the lower area of the top three leaves and aboveground dry weight per shoot at 25 cm×23 cm spacing in soil fertility 2 may be attributed to limited N supply and/or N uptake. A recent study reported low N concentrations in shoots due to dilution as a result of more biomass production [43], which suggests that rice plants seeded at wider spacing might need more fertilizer to obtain high yield than those subjected to dense spacing. More fertilizer is needed not only to improve productive tiller percentage at wide spacing but also to increase N concentrations in plants and to improve dry weight accumulation and grain yield per shoot. Nevertheless, we think that the application of extra N to compensate for the reduced grain yield due to low planting density is not a good option because of potential environmental problems and increased cost for rice farmers.

In the on-farm demonstration experiments, mechanical hill seeding of rice at 25 cm×17 cm spacing had equal or higher grain yield than transplanted rice, indicating that this spacing was the optimum seeding density for high-yielding hybrid rice varieties. Hill-seeded rice had more panicles per unit area and less spikelets per panicle compared with transplanted rice.
